# Selected Oxidative Stress Markers in Colorectal Cancer Patients in Relation to Primary Tumor Location—A Preliminary Research

**DOI:** 10.3390/medicina56020047

**Published:** 2020-01-21

**Authors:** Karolina Janion, Elżbieta Szczepańska, Ewa Nowakowska-Zajdel, Joanna Strzelczyk, Angelika Copija

**Affiliations:** 1 Department of Nutrition-Related Disease Prevention, Department of Metabolic Disease Prevention, School of Health Sciences in Bytom, Medical University of Silesia in Katowice, 40-055 Katowice, Poland; ewanz@onet.eu (E.N.-Z.); ang.c@wp.pl (A.C.); 2 Department of Human Nutrition, School of Health Sciences in Bytom, Medical University of Silesia in Katowice, 40-055 Katowice, Poland; e.szczepanska@sum.edu.pl; 3 Department of Clinical Oncology, No. 4 Provincial Specialist Hospital, 41-902 Bytom, Poland; 4 Department of Medical and Molecular Biology, School of Medical Sciences in Zabrze, Medical University of Silesia in Katowice, 40-055 Katowice, Poland; asia.strzelczyk@gmail.com

**Keywords:** colorectal cancer, oxidative stress, lipid peroxidation, right/left sided tumor

## Abstract

*Background and objectives:* Ample evidence indicates that oxidative stress, including complex lipid peroxidation processes, may play a significant role in the pathogenesis of colorectal cancer. The goal of this study was to evaluate selected oxidative stress markers in patients with colorectal cancer depending on some clinical features, with particular attention paid to the location of the primary tumor. *Materials and Methods:* The study was conducted on a group of 66 patients with colorectal cancer. The study consisted of two stages. The first stage involved the analysis of medical records; the second consisted of determining selected oxidative stress markers by measuring malondialdehyde as well as total oxidant and antioxidant status. *Results:* Of all patients, 43 (65.15%) had colon cancer, of whom 30 (69.77%) had a tumor on the left side and 13 (30.23%) had a tumor on the right side of the colon. Of all the patients, 23 (34.85%) had rectal cancer. The mean total oxidant and antioxidant status was 809.76 (SD ± 392.65) µmol/L and 253.19 (233.33–310.66) µmol/L, respectively. The mean malondialdehyde serum level was 2478.04 (SD ± 1397.05) ng/mL. The mean malondialdehyde serum concentration in patients with primary tumors located on the right side was higher in a statistically significant way compared with the remaining patients. *Conclusions:* It was demonstrated that the intensity of lipid peroxidation processes is correlated with the development of colorectal cancer, particularly on the right side. The results should be interpreted rather cautiously due to certain limitations of the study.

## 1. Introduction

According to the GLOBOCAN 2018 data, colorectal cancer (CRC) is the fourth most common malignancy in the world [1]. More than a half of all cases are diagnosed in countries with a high or very high human development index (HDI). More and more new cases are being noted in countries of low income; this is associated with a shift in eating habits towards greater consumption of animal fat and simple sugars. The prevalence of CRC increases with age and is higher in males [2,3]. Moreover, women more often present tumors on the right side of the colon (so-called proximal tumors), which are linked with a more aggressive course when compared with lesions located on the left side of the colon (so-called distal tumors) [4].

The differences between proximal and distal colon cancer were described at the beginning of the 1990s, with Bufill suggesting different biological pathways for these cancers [5]. Diverse embryological origin was suggested as one of the causes. The left side of the colon derives from the embryonic hindgut, whereas its right side derives from the midgut. Some authors therefore postulate that left- and right-sided colon cancers should be viewed as two distinct disease entities [6,7]. In advanced neoplasia of the colon, distal tumors are characterized by their polypoid structure and are easier to detect in colonoscopy compared with flat proximal tumors. This is why patients with a right-sided location of the primary tumor are more often diagnosed at an advanced stage, which is an additional unfavorable prognostic factor [4,6].

The differences between left- and right-sided colon cancer may also result from diverse genetic compositions and lifestyles. In right-sided colon cancer, high microsatellite instability (MSI), BRAF mutation and hereditary nonpolyposis colorectal cancer (HNPCC) are more common. The left-sided disease, in turn, is associated with chromosomal instability, K-ras mutation and familial adenomatous polyposis (FAP) [4,8].

Moreover, dietary factors have variable influence on CRC development. It has been shown that high consumption of simple sugars increases the probability of right-sided colon carcinoma in women and is associated with higher probability of rectal disease in men. Overconsumption of fats, on the other hand, especially trans fats, results in greater likelihood of developing proximal colon cancer [4]. Ferrucci et al. observed that consumption of processed red meat was associated with an increased risk of left-sided colon cancer, while consumption of highly grilled meat was positively correlated with rectal cancer [9]. Other works suggest that high serum vitamin D levels and high consumption of dietary calcium are negatively correlated with left-sided colon cancer, but these results are unclear [10].

So, the pathogenesis of CRC is complex and is a result of family burden, genetic predisposition and environmental factors, including diet. A number of authors also point to oxidative stress as a significant factor in the development of this cancer. Oxidative stress is a disruption of the body’s homeostasis resulting from the activity of reactive oxygen species (ROS). Due to the disproportion between compounds acting as pro- and antioxidants, a range of negative reactions take place that consequently damage cellular structures, including lipids [11,12].

Lipid peroxidation is a highly complex process that consists of a range of consecutive, and at the same time overlapping, reactions. Final products of lipid oxidation include 4-hydroxynonenal (4-HNE) and malondialdehyde (MDA). MDA is highly reactive, capable of inhibiting enzymes that protect cells against the harmful effects of oxidative stress; it also has cytotoxic and carcinogenic properties [13,14]. Its blood serum level is a measure of lipid peroxidation. Total oxidant status (TOS) and total antioxidant status (TAS) are parameters used to measure the general oxidative and antioxidant status of the body, respectively [15].

Investigating MDA and oxidative stress markers depending on the location of the tumor (left or right side of the colon) seems interesting as data in this area are not available.

The goal of this study is to evaluate selected oxidative stress markers in CRC patients depending on some clinical features, especially the location of the primary tumor.

## 2. Materials and Methods

### 2.1. Design, Patients and Samples

The study group consisted of 66 CRC patients (25 women and 41 men) hospitalized at the Department of Clinical Oncology of the Provincial Specialist Hospital No. 4 in Bytom, Poland. Patients above the age of 18 years with histologically confirmed CRC, regardless of the clinical stage, and patients at least 4 weeks after surgery were included in the study group to start oncological treatment (systemic therapy as adjuvant or palliative care). All examined patients had not received systemic therapy before. Patients with another history of cancer (apart from squamous cell skin cancer), patients with cancer-associated cachexia, patients that required long-term immunosuppression, and patients diagnosed with infection (tuberculosis, hepatitis B or C, HIV or AIDS) were excluded from the investigation. Recruitment of respondents lasted from June 2017 to December 2018. During hospitalization, serum samples (3 cm^3^) were collected from examined patients. Patients’ blood samples were collected before the initiation of systemic therapy. The tests were performed using blood collected in the morning from fasting patients (approximately 8–10 h after the last meal). Blood was immediately centrifuged, and serum was stored frozen at −80 °C until analysis.

The study consisted of two stages. The first stage involved the analysis of medical records, with the assessment of the CRC stage according to the TNM Classification of Malignant Tumors, in accordance with the guidelines of the American Joint Committee on Cancer (8th Edition Cancer Staging Manual 2015), tumor location (right/left side of the colon and rectum), histological malignancy grade, comorbidities (cardiovascular diseases, type 2 diabetes mellitus, obesity) and used drugs. The second stage consisted of the determination of oxidative stress markers by measuring MDA as well as TOS and TAS levels. This study was conducted following the Declaration of Helsinki and good clinical practice guidelines. The investigation was approved by the Ethics Committee of the Medical University of Silesia in Katowice (Resolution No KNW/0022/KB1/43/17 of 30 May 2017). Patient participation was voluntary. All patients expressed written informed consent to participate in the project.

### 2.2. MDA, TOS and TAS Determinations

MDA (Cloud-Clone Corp., cat. No CEA597Ge, Houston, TX, USA) was assayed using enzyme-linked immunosorbent assay (ELISA), whereas TOS and TAS were determined using the following reagent kits: PerOx (Immunodiagnostik, cat. No KC 5100, Bensheim, Germany) and ImAnOx (Immunodiagnostik, cat. No KC 5200, Bensheim, Germany), in accordance with the methodology provided by the manufacturer. The assays have high sensitivity and excellent specificity for the detection of MDA serum concentration, TOS and TAS levels. The absorbance readings were recorded at waves of 450 nm and then calibrated according to the standard curve for MDA serum concentrations in ng/mL and for TOS and TAS levels in μmol/L. All samples were run in duplicate.

### 2.3. Statistical Analysis

The data were collected in Microsoft Excel 2010, and the statistical analysis was performed in Statistica 13.3 (TIBCO Statistica™, StatSoft, Kraków, Poland). Firstly, it was checked whether quantitative variables were in agreement with normal distribution using the Shapiro-Wilk W test and normality diagrams. Quantitative variables were presented as arithmetic means with standard deviation (SD) for near-to-normal distribution or median values with interquartile ranges (from P25 to P75) for non-normal distribution. The quantitative variables with distribution nearing normal distribution were compared using the Student’s t-test (for two samples), and the quantitative variables with non-normal distribution were analyzed with a non-parametric Mann-Whitney U test (for two samples). To compare more than two samples, the Kruskal–Wallis ANOVA was applied; a two-sided test with Bonferroni correction was used for post hoc analysis. For all analyses, the value of *p* < 0.05 was considered statistically significant.

## 3. Results

### 3.1. Clinical Parameters of the Patients

The oxidative stress markers assayed from the serum were analyzed for 66 patients. Of all these patients, 43 (65.15%) had colon cancer, of whom 30 (69.77%) had a tumor on the left side and 13 (30.23%) had a tumor on the right side of the colon. Of all the patients, 23 (34.85%) had rectal cancer. The characteristics of all the patients are presented in Table 1.

### 3.2. Oxidative Stress and Primary Tumor Location

The higher mean TOS level was observed in patients with right-sided tumor location compared with patients with left-sided tumor location. Interestingly, the mean TAS level was also highest in patients with right-sided tumor location. In the investigated patients, there were no differences in the TOS level (Kruskal–Wallis ANOVA; *p* = 0.790) and the TAS level (Kruskal–Wallis ANOVA; *p* = 0.888) in the serum and the primary tumor location in the colon (left or right side) or rectum (Table 2).

**Figure 1:**
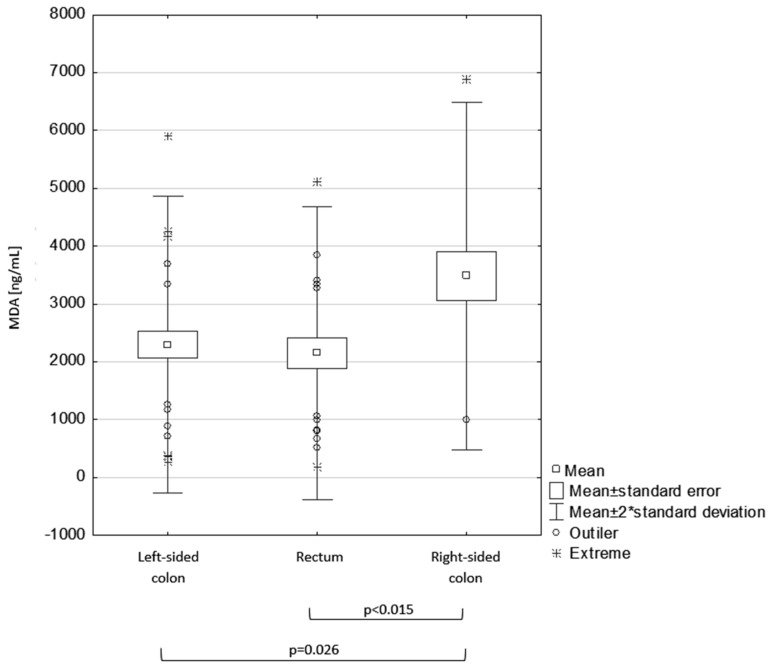
Malondialdehyde (MDA) serum concentration and primary tumor location.

In turn, MDA serum concentration was highest in patients with right-sided tumor location. There were statistically significant differences in MDA serum concentration and the primary tumor location in the colon (left or right side) or rectum (Kruskal–Wallis ANOVA; *p* = 0.026). A two-sided post hoc test with Bonferroni correction revealed statistically significant differences in MDA levels in the left and right side of the colon (*p* = 0.026), as well as in the right side of the colon and rectum (*p* < 0.015) (Figure 1, Table 2).

The mean MDA serum concentration in patients with primary tumors located on the right side was higher in a statistically significant way compared with the remaining patients (Figure 1, Table A1).

### 3.3. Oxidative Stress and Clinical Stage of Disease

The highest mean TOS level was observed in stage IV and stage I of the disease, respectively. The mean MDA serum concentration was highest in patients with stage IV cancer.

Moreover, there were no differences in the results of the TOS level (Kruskal–Wallis ANOVA; *p* = 0.310), the TAS level (Kruskal–Wallis ANOVA; *p* = 0.539) and MDA serum concentration (Kruskal–Wallis ANOVA; *p* = 0.552) in the serum at different clinical stages of the disease (Table 3).

### 3.4. Oxidative Stress and Comorbidities

Higher mean TOS level and MDA serum concentrations were observed in patients with cardiovascular diseases (CVD) compared with patients without CVD. However, there were no significant differences in the TOS level (Student’s t-test; *p* = 0.07), TAS level (Mann–Whitney test; *p* = 0.50) and MDA serum concentration (Student’s t-test; *p* = 0.21) between the group of patients with CVD and without CVD (Table 4).

In turn, higher mean TOS level, TAS level and MDA serum concentration were observed in patients with type 2 diabetes mellitus (T2DM). There were differences in the MDA serum concentration (Student’s t-test; *p* = 0.04) between the group of patients with T2DM and without T2DM, but there were no significant differences in the TOS level (Student’s t-test; *p* = 0.69) or the TAS level (Mann–Whitney U test; *p* = 0.596) between the group of patients with T2DM and without T2DM (Table 4).

There were no differences in the TOS level (Student’s t-test; *p* = 0.97), the TAS level (Mann–Whitney U test; *p* = 0.42) and MDA serum concentration (Student’s t-test; *p* = 0.09) in patients with and without obesity (Table 4).

### 3.5. Oxidative Stress and Age

**Figure 2:**
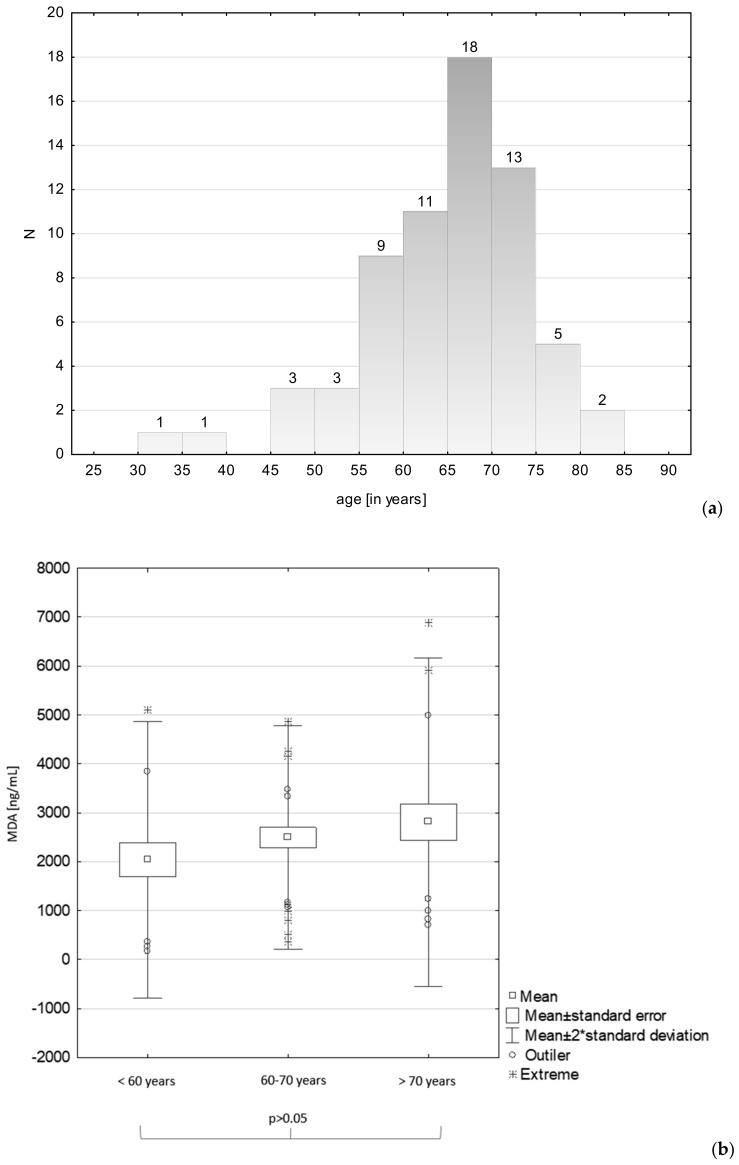
Age distribution in the study group (**a**) and MDA serum concentration and patients’ age (**b**).

The population was diversified in terms of age (Figure 2a). The population was divided into three groups (>60 years, 60–70 years, >70 years) due to the high heterogeneity of the group, including comorbidities in patients older than 70 years and the occurrence of progressive aging processes compared with patients from younger age groups.

The intensity of lipid peroxidation was evaluated taking into account patients’ age (Figure 2b). Based on the Kruskal–Wallis ANOVA, no statistically significant differences were found in the MDA serum concentration and age (*p* = 0.35), but the mean MDA serum concentration was the highest in the oldest patients (i.e., those over 70 years of age).

## 4. Discussion

Numerous recent studies have focused on the role of oxidative stress in neoplastic diseases, including CRC. The findings obtained thus far suggest that more intense oxidative stress and lower antioxidant protection mechanisms may play a significant role in the pathogenesis of CRC. Various authors have observed an increase in the MDA level in CRC patients compared with controls [16,17,18,19]. Only a few studies conducted so far present a detailed assessment of the intensity of lipid peroxidation processes in CRC patients depending on the co-occurrence of metabolic diseases, clinical stage of cancer or primary tumor location. The present study involved the analysis of selected oxidative stress markers in relation to these clinical features. Moreover, it was attempted in order to evaluate the intensity of oxidative stress depending on primary tumor location (right/left side of the colon and rectum).

Right-sided colon cancer (a tumor developing in the caecum, ascending colon, hepatic flexure and two-thirds of the proximal transverse colon) is linked with worse prognosis compared with left-sided colon cancer (a tumor developing in the remaining one-third of the transverse colon, splenic flexure, descending colon and sigmoid). The right-sided location is characterized by adverse prognostic factors, such as greater tumor size or higher malignancy grade [4,7]. The present study revealed statistically significant differences in MDA serum concentration and primary tumor location. It was found that the right-sided location is linked with enhanced lipid peroxidation processes compared with the left side and the rectum. However, TOS and TAS levels did not vary significantly (*p* > 0.05). Similar results have been reported by Wu et al. concerning plasma TOS and TAS levels. These authors did not observe any statistically significant differences between TOS and TAS levels and primary tumor location in the colon and rectum (*p* > 0.05), but they did not include the division into right- and left-sided colons [15].

Researchers are also investigating a potential relationship between oxidative stress markers and clinical stages of cancer [15,16]. In our study, serum MDA levels reached the highest values in patients with stage IV cancer, but these findings were not statistically significant (*p* > 0.05). Rašić et al. noted similar results. Their study demonstrated statistically significant differences in serum MDA levels and stage II and III of the disease (*p* < 0.01) [20]. However, Wu et al. observed no statistically significant differences in TOS and TAS levels and the clinical stage of the cancer (*p* < 0.05) [15]. Similar results were obtained in the present study, but due to small sample sizes in subgroups with stage I (*n* = 4) and stage II carcinoma (*n* = 15), these patients were evaluated jointly.

The development of CRC is strictly correlated with lifestyle. Non-balanced diet, alcohol use, smoking tobacco and a sedentary lifestyle are factors that increase the risk of CRC and metabolic diseases [21]. The role of oxidative stress in the development of metabolic syndrome has been confirmed, but mechanisms responsible for its occurrence are complex and have not been fully identified [22]. In patients with T2DM, chronic hyperglycaemia contributes to enhanced production of intracellular glucose and ROS. Long-term cell exposure to the activity of ROS exhausts the possibilities of neutralizing harmful compounds, which distorts the peroxidative–antioxidant balance of the organism [23]. An additional factor that is conductive to a change of balance is concomitant abdominal obesity. Visceral fat is capable of proinflammatory cytokine production, for instance IL-1, IL-6 and tumor necrosis factor α (TNF-α), thus creating an environment that favors ROS production [24]. Other factors contributing to oxidative stress in obesity include lipid metabolism disorders, endothelial dysfunction, deficiency of certain vitamins and minerals, and diet [25].

The present study investigated oxidative stress in relation to CVD, T2DM and obesity. It was demonstrated that lipid peroxidation processes are more intense in CRC patients with concomitant T2DM than in patients without it (*p* = 0.04). Huang et al. also noted higher serum MDA levels in T2DM patients compared with controls and patients with pre-diabetes [26]. The same outcomes have been reported by Jiménez-Osorio et al. These authors showed a decline in TAS levels in patients with uncompensated T2DM compared with controls [27]. In the present study, levels of TOS were higher and levels of TAS were lower in patients with T2DM, compared with patients with no carbohydrate metabolism disorders. These data should be treated with caution as the number of patients in the T2DM subgroup was small. Furthermore, there is some evidence for enhanced lipid peroxidation in patients with T2DM and concomitant complications. For instance, Djindjica et al. observed high MDA levels in patients with T2DM and stable ischaemic heart disease [28]. Moreover, Akila et al. noted markedly higher MDA levels in elderly patients with T2DM and hypertension, compared with elderly patients with T2DM or hypertension alone and elderly patients without any comorbidities. They additionally underlined that complications of T2DM in hypertensive patients are associated with a long history of these diseases, which leads to the accumulation of free radicals and lipid peroxidation products [29]. In the present study, patients with CRC and a positive history of CVD had higher MDA and TOS levels and lower TAS levels than patients without CVD, but these values were not statistically significant (*p* > 0.05). The same observations were noted in obese patients with CRC compared with non-obese individuals. The results are in line with the reports of other authors. Sankhla et al. also observed higher serum MDA levels in obese individuals compared with those with normal body weight. Moreover, the intensity of lipid peroxidation was most evident in patients with abdominal obesity [30].

Furthermore, intensive studies aiming to determine the relationship between developing CRC, obesity and gut microbiota composition are underway. However, due to the progressive dynamics of the composition and metabolic bacteria activity under the effects of various environmental factors, finding this relationship is difficult. Nevertheless, metabolic activity of certain microorganisms leads to the formation of mutagenic and carcinogenic compounds in the large bowel. It has been shown that Enterococcus faecalis is capable of producing hydroxyl radical, which has an adverse effect on intestinal epithelium and may be a source of oxidative stress in CRC patients [11].

Also, a number of authors suggest that ageing processes are strictly associated with enhanced lipid oxidation, but available data are inconsistent. Several studies have indicated an increase in MDA levels with age [29,31,32]. Akila et al. found that MDA levels are higher in healthy individuals aged 60–75 years compared with healthy younger groups (20–32 years) [28]. Fasna et al. also proved that ageing processes are positively correlated with high concentrations of lipid peroxidation products, and their results were statistically significant [30]. Our study did not demonstrate statistically significant differences between MDA levels and age (*p* > 0.05). Also, lower serum MDA levels were noted in patients younger than 60 years of age compared with those over 70 years of age. When interpreting these results, the heterogeneity of the study group in terms of concomitant diseases and nutritional status, which is indirectly associated with varied BMI values, must be kept in mind.

To sum up, there is evidence that oxidative stress is an important factor in the development of various diseases, including CRC. Current knowledge does not allow unambiguous conclusions as to whether oxidative stress initiates carcinogenesis or is a consequence of neoplastic transformation [33]. The complexity of the processes makes further studies necessary to specify the role of oxidative stress in CRC. It seems interesting to find an answer to the question of whether oxidative stress is more strongly correlated with right-sided CRC, which is also linked with poorer prognosis. Further research should be conducted on larger populations and account for environmental factors, including nutrition, ethylene alcohol consumption and smoking status. Individual dietary factors will have variable effects on the development of CRC, e.g., right-sided tumors are more common in patients with predominant eating habits associated with high simple sugar and fat consumption [4]. A potential relationship between oxidative stress, diet and CRC seems to be significant enough to be taken into account in planning primary and secondary prevention as well as supportive treatment of CRC.

## 5. Conclusions

Oxidative stress may play a significant role in CRC, but the mechanisms responsible for its occurrence remain unclear. The understanding of CRC risk factors may improve the development of personalized medicine. Our study demonstrated differentiation in the intensity of lipid peroxidation processes in relation to the location of the primary tumor. Since the anchor point for molecular targeted therapy is still being searched for, studies on these issues seem to be important. The results should be interpreted rather cautiously due to the limitations of the study. Further research is needed to verify these findings.

## 6. Limitations

A relatively small sample size (leading to the lack of multivariate analysis, which was not possible due to small sample size), no control group and the lack of environmental factors in the analysis, e.g., diet, dietary supplements, ethylene alcohol consumption and tobacco smoking, are the limitations of this study.

## Figures and Tables

**Table 1:** Clinical characteristics of the study group, including primary tumor location.

Clinical Features	Primary Tumor Location			Total (*n* = 66)
	Left-Sided Colon (*n* = 30)	Right-Sided Colon (*n* = 13)	Rectum (*n* = 23)	
Female Male	11 (36.67%)	7 (53.85%)	7 (39.43%)	25 (37.88%)
	19 (63.33%)	6 (46.15%)	16 (69.57%)	41 (62.12%)
Age [years]	68.00	69.00	66.00	65.50
	(59.00–73.00)	(61.00–74.00)	(57.00–73.00)	(60.00–73.00)
BMI [kg/m2]	25.51	27.98	26.50	26.34
	(SD ± 4.34)	(SD ± 4.04)	(SD ± 3.86)	(SD ± 4.16)
Normal body weight (BMI value: 18.5–24.9)	16 (53.33%)	4 (30.77%)	10 (43.48%)	30 (45.45%)
Overweight (BMI value: 25.0–29.9)	12 (40.00%)	4 (30.77%)	7 (30.43%)	23 (34.85%)
Obesity (BMI value: ≥30.0)	2 (6.67%)	5 (38.46%)	6 (26.09%)	13 (19.70%)
CVD 1—Yes	19 (63.33%)	7 (53.85%)	12 (52.17%)	38 (57.58%)
CVD—No	11 (36.67%)	6 (46.15%)	11 (47.83%)	28 (42.42%)
T2DM 2—Yes	3 (10.00%)	1 (7.69%)	4 (17.39%)	8 (12.12%)
T2DM—No	27 (90.00%)	12 (92.31%)	19 (82.61%)	58 (87.88%)
CS 3 I	0 (0.00%)	0 (0.00%)	4 (17.39%)	4 (6.06%)
CS II	7 (23.34%)	3 (23.08%)	5 (21.74%)	15 (22.73%)
CS III	13 (43.33%)	3 (23.08%)	6 (26.09%)	22 (33.33%)
CS IV	10 (33.33%)	7 (53.84%)	8 (34.78%)	25 (37.88%)
Gx	4 (13.33%)	1 (7.69%)	6 (26.09%)	11 (16.66%)
G1	4 (13.33%)	1 (7.69%)	3 (13.04%)	8 (12.12%)
G2	18 (60.01%)	7 (53.85%)	13 (56.52%)	38 (57.58%)
G3	4 (13.33%)	4 (30.77%)	1 (4.35%)	9 (13.64%)

^1^ CVD—cardiovascular diseases; ^2^ T2DM—type 2 diabetes mellitus; ^3^ CS—clinical stage.

**Table 2:** Oxidative stress markers taking into account the primary tumor location.

Primary Tumor Location	TOS [µmol/L]	*p*	TAS [µmol/L]	*p*	MDA [ng/mL]	*p*
Left-sided colon (*n* = 30)	827.66 (SD ± 443.80)	0.790	257.60 (235.60–294.23)	0.888	2294.04 (SD ± 1283.00)	0.026
Right-sided colon (*n* = 13)	842.78 (SD ± 323.54)		278.76 (231.59–337.10)		3481.63 (SD ± 1503.26)	
Rectum (*n* = 23)	767.75 (SD ± 368.58)		269.06 (236.13–278.47)		2150.80 (SD ± 1269.15)	
Total (*n* = 66)	809.76 (SD ± 392.65)	-	253.19 (233.33–310.66)	-	2478.04 (SD ± 1397.05)	-

**Table 3:** Oxidative stress markers taking into account the clinical stage of the disease.

TNM Classification	TOS [µmol/L]	*p*	TAS [µmol/L]	*p*	MDA [ng/mL]	*p*
CS ^1^ I and II (*n* = 19)	694.32 (SD ± 407.92)	0.310	268.99 (234.26–362.35)	0.539	2541.23 (SD ± 1722.50)	0.552
CS III (*n* = 22)	829.62 (SD ± 403.33)		251.16 (233.53–272.46)		2239.99 (SD ± 995.50)	
CS IV (*n* = 25)	880.02 (SD ± 366.54)		251.49 (235.60–290.49)		2639.50 (SD ± 1453.03)	
Total (*n* = 66)	809.76 (SD ± 392.65)	-	253.19 (233.33–310.66)	-	2478.04 (SD ± 1397.05)	-

^1^ CS—clinical stage.

**Table 4:** Oxidative stress markers taking into account comorbidities.

Comorbidities		TOS (µmol/L)	*p*	TAS (µmol/L)	*p*	MDA (ng/mL)	*p*
CVD ^1^	No (*n* = 28)	708.11 (SD ± 429.69)	0.07	256.57 (225.85–300.58)	0.500	2223.42 (SD ± 1246.08)	0.21
	Yes (*n* = 38)	884.67 (SD ± 350.03)		253.20 (237.20–318.27)		2665.65 (SD ± 1486.76)	
T2DM ^2^	No (*n* = 58)	802.56 (SD ± 396.85)	0.69	251.49 (233.33–294.23)	0.596	2345.16 (SD ± 1359.78)	0.04
	Yes (*n* = 8)	861.97 (SD ± 381.57)		264.51 (235.73–355.54)		3441.44 (SD ± 1363.84)	
Obesity	No (*n* = 53)	810.59 (SD ± 400.75)	0.97	254.90 (232.53–294.23)	0.420	2332.07 (SD ± 1394.99)	0.09
	Yes (*n* = 13)	806.39 (SD ± 373.02)		251.49 (247.22–310.66)		3073.17 (SD ± 1288.99)	
Total (*n* = 66)		809.76 (SD ± 392.65)	-	253.19 (233.33–310.66)	-	2478.04 (SD ± 1397.05)	-

^1^ CVD—cardiovascular diseases; ^2^ T2DM—type 2 diabetes mellitus.

## Appendix A

**Table A1:** MDA serum concentration and primary tumor location.

Primary Tumor Location	Mean	SD
Left-sided colon (*n* = 30)	2294.04	±1283.00
Right-sided colon (*n* = 13)	3481.63	±1503.26
Rectum (*n* = 23)	3481.63	±1269.15
Total (*n* = 66)	2478.04	±1397.05
